# Surveillance of healthcare associated infections in Dutch nursing homes

**DOI:** 10.1186/2047-2994-4-S1-P275

**Published:** 2015-06-16

**Authors:** K Halonen, A Haenen, E Smid, S de Greeff

**Affiliations:** 1 Epidemiology and Surveillance, National Institute of Public Health and Environment, Netherlands

## Introduction

Elderly residents of nursing homes have a higher risk for an infection and are at risk due to underlying chronic illnesses, dependence on care, sharing of facilities and living in a close contact with others. Still, systematic surveillance on prevalence of healthcare-associated infections (HAIs) in this population is scarce.

## Objectives

The aim of this study was to determine the prevalence of HAIs in nursing homes in the Netherlands in order to identify the burden of and risk factors for infection.

## Methods

Since 2009 the Dutch national surveillance network for HAIs in nursing homes (SNIV) organizes biannual crossectional prevalence surveys. For all residents in participating nursing homes baseline characteristics, information on the presence of gastroenteritis, lower respiratory tract infection (LTI), urinary tract infection (UTI), bacterial conjunctivitis, bloodstream infection and antibiotic use were collected from 2010 till 2014. Coding of infections was based on clinical definitions.

## Results

On average 25 nursing homes participated per survey (range: 3-49). Most nursing homes participated for several years. In total 17241 residents were included and 534 HAIs were registered. The overall prevalence of HAIs was 3.1% (95%CI: 2.8-3.4). Most HAIs occurred among residents of rehabilitation units (4.2%, 95%CI: 3.3-5.3). The most common infections were the UTIs (2.0%, 95%CI: 1.8-2.2) and LTIs (0.7%, 95%CI: 0.5-0.8). 18.9% of the UTIs were found among residents having a catheter. For the UTIs a significant decrease in prevalence over the years was observed from 3.0 and 3.1% in 2010 and 2011 respectively, to 1.6% in 2014 (p<0.01). Since 2010 the monitored prevalence of residents on antibiotic therapy during the survey significantly decreased from 8.1% in 2010 to 4.1% in 2014 (p<0.01).

## Conclusion

Repeated surveys support evaluation of local infection control policies, interventions and guides national policy making. Based on 5 years of biannual prevalence studies a decreasing trend in the prevalence of HAI’s and particularly urinary tract infections was observed as well as a decline in percentage of residents having antibiotics. This may suggest improved infection prevention measures in the participating nursing homes. Further, in depth analysis is needed to study factors associated with the decrease in HAI and to possibly identify best practices.

## Disclosure of interest

None declared.

